# Regulation of Synaptic NMDA Receptor Activity by Post-Translational Modifications

**DOI:** 10.1007/s11064-025-04346-6

**Published:** 2025-03-03

**Authors:** Emanuel Tahiri, Elisa Corti, Carlos B. Duarte

**Affiliations:** 1 CNC-UC-Center for Neuroscience and Cell Biology, University of Coimbra, Coimbra, Portugal; 2 CiBB - Centre for Innovative Biomedicine and Biotechnology, University of Coimbra, Coimbra, Portugal; 3 III- Institute for Interdisciplinary Research, University of Coimbra, Coimbra, Portugal; 4 Department of Life Sciences, University of Coimbra, Coimbra, Portugal; 5 Center for Neuroscience and Cell Biology, Faculty of Medicine, University of Coimbra, Rua Larga, Coimbra, 3004-504 Portugal

**Keywords:** Glutamatergic synapses, NMDA receptors, Synaptic plasticity, Phosphorylation, Ubiquitination, Palmitoylation

## Abstract

NMDA receptors for the neurotransmitter glutamate are widely distributed in the central nervous system, playing important roles in brain development, function and plasticity. Alterations in their activity are also important mediators in neuropsychiatric and neurodegenerative disorders. The different NMDA receptor subunits (GluN1, GluN2A-D and GluN3A, B) share a similar structure and membrane topology, with an intracellular C-terminus tail responsible for the interaction with proteins important for the trafficking of the receptors, and to control their surface distribution and signalling activity. The latter sequence varies among subunits but consistently contains the majority of post-translational modification sites on NMDA receptors. These modifications, including phosphorylation, ubiquitination, and palmitoylation, regulate interactions with intracellular proteins. Differences in the amino acid sequence between NMDA receptor subunits lead to a differential regulation by post-translational modifications. Since NMDA receptors are formed by oligomerization of different subunits, and each subunit is regulated in a specific manner, this creates multiple possibilities for regulation of these receptors, with impact in synaptic function and plasticity. This review addresses the diversity of mechanisms involved in the post-translational modification of NMDA receptor subunits, and their impact on the activity and distribution of the receptors, as well as their function in nerve cells.

## Introduction

The N-methyl-D-aspartate receptors (NMDAR) are widely distributed in the brain [1–3], playing important roles in synaptic development, synaptic transmission and in plasticity [4–6]. Dysregulation of the activity of NMDAR is also associated with different types of diseases of the nervous system [5, 6]. These receptors are expressed not only in neurons but also in glial cells and endothelium cells in the brain [7].

NMDAR are ligand-gated ion channels, formed by the assembly of two GluN1 obligatory subunits and two subunits belonging to the GluN2 family (GluN2A to GluN2D). Alternatively, NMDAR may also be formed by oligomerization of GluN1 together with GluN3 (GluN3A, 3B) subunits [4–6, 8]. GluN1 subunits contain the glycine/D-serine binding site, while GluN2 subunits are responsible for the interaction with glutamate. GluN3 subunits also bind glycine and, therefore, the receptors containing this subunit together with GluN1 are activated by glycine alone [5, 6, 8]. Activation of NMDAR receptors containing GluN1 and GluN2 subunits requires interaction with the two co-agonists, glutamate and glycine/D-serine, and depolarization of the membrane, allowing the entry of Na^+^ and Ca^2+^ [4, 9].

The expression of GluN2 subunits is differentially regulated during development and in different brain regions. In rodents, GluN2B and Glu2D are highly expressed early in development, but the expression of the former subunit becomes restricted to the forebrain in the adult [10–12]. On the other hand, the expression of GluN2A starts at birth and increases progressively to become widely expressed throughout the CNS. Accordingly, during development of the cerebral cortex and hippocampus, GluN2B is partly replaced by GluN2A in the composition of NMDAR [10, 13, 14]. The expression of GluN2C is also developmentally regulated, and this subunit is more abundant in the adult cerebellum, where it replaces GluN2B found at earlier stages of development [12]. Depending on their subunit composition, the NMDAR exhibit distinct electrophysiological and signalling properties. Thus, the observed switch from GluN2B- to GluN2A-containing NMDAR observed during development results in a decreased Ca^2+^-permeability and charge transfer, as well as in a faster rate of deactivation, rise and decay times [15].

The abundance and subunit composition of synaptic NMDAR is controlled by the balance between the rate of synthesis and assembly of new receptors, which take place in the Golgi apparatus at the soma or in dendritic Golgi outposts, their dendritic transport, the rate of receptor delivery to the plasma membrane, their lateral diffusion, and the internalization, recycling and degradation [15–17]. In addition, the surface distribution of NMDAR is determined by the equilibrium in the exchange between the synaptic and extrasynaptic pools of the receptor. More than one third of the NMDAR are located extrasynaptically, being the remaining concentrated within the postsynaptic region [18–21]. Analysis of the dynamics of NMDAR showed that GluN2A-containing NMDAR display a lower diffusion coefficient when compared with receptors containing GluN2B subunits, both in the synaptic and extrasynaptic regions [22], although distinct results were obtained depending on the methodology used to assess the receptor dynamics [23]. However, GluN2A- containing NMDAR are more stable at the synapse when compared with receptors with GluN2B subunits [22, 23]. Compared to NMDAR containing GluN2A or GluN2B subunits, receptors with GluN3A subunits display the lowest residence time at the synapse and the highest synaptic-extrasynaptic exchange rate [23].

At the synapse, NMDAR and their intracellular scaffold proteins are present in nanodomains which were proposed to align with presynaptic sites of neurotransmitter release [24]. In vivo studies also showed that NMDAR can be found in two distinct populations: complexes with approximately 0.8MDa and supercomplexes with about 1.5 MDa [25, 26]. This synaptic organization provides a high efficiency in synaptic transmission and in synaptic plasticity [27, 28]. GluN2A- and GluN2B-containing NMDAR show a largely non-overlapping nanoscale distribution in hippocampal synapses, pointing to the differential contribution of specific sorting mechanisms depending on the receptor composition [27, 28]. In addition, the nanoclusters of GluN2A-containing NMDAR are more abundant and larger than those with GluN2B subunits. However, the molecular mechanisms that determine the differential distribution of GluN2A- and GluN2B-containing NMDAR at the synapse remain to be elucidated. Extrasynaptic NMDAR are also distributed in small nanodomains that are more abundant than their synaptic counterparts [28].

## Topology of NMDA Receptor Subunits and Receptor Anchoring at the Synapse

All NMDAR subunits are formed by the assembly of four subunits which share a similar structure. Their extracellular region includes an extracellular N-terminal domain (NTD), also referred to as amino-terminal domain (ATD), which is relevant in the assembly of the receptors, their trafficking and regulation, and a ligand-binding domain (LBD), also named agonist binding domain (ABD). The transmembrane region of each subunit comprises three transmembrane domains (M1, M3 and M4), and a re-entrant loop (M2) that forms the pore of the receptor. Finally, the intracellular C-terminal domain (CTD) of NMDAR subunits shows great diversity when the different NMDAR subunits are compared, both in their amino acid sequence and in length (50 and 650 amino acids in GluN1 and in GluN2B, respectively). The CTD of NMDAR subunits plays a key role in the interaction with a large number of intracellular proteins and in the regulation of the receptors, acting as molecular hubs for the integration of regulatory and signalling events [4, 29].

The distribution of NMDAR between the synaptic and extrasynaptic compartments is determined by proteins belonging to the membrane-associated guanylate kinase (MAGUK) family, in particular PSD-95 (postsynaptic density-95), PSD-93, SAP97 (synapse associated protein-97; also known as DLG 1) and SAP102 [30–33]. These proteins are characterized by the presence of three conserved PDZ (post-synaptic density-95/discs-large/zona-occludens-1) domains, one SH3 domain (SRC Homology 3 Domain) and a GK (guanylate kinase-like) modular domain that is catalytically inactive [34–36]. Both GluN2A and GluN2B possess a conserved PDZ-binding motifs (Glu-Ser[Asp/Glu]Val) which allow direct high affinity interaction with MAGUK family members. Protein-protein interaction studies performed in extracts from the rat hippocampus showed a preferential binding of GluN2A to PSD-93, PSD95 and SAP97, while GluN2B complexes were enriched in SAP102 and PSD-95 [37]. Disruption of the interaction of GluN2A- and GluN2B-containing NMDAR with MAGUKs (membrane-associated guanylate kinase proteins) using biomimetic competing ligands showed a differential regulation by the interaction of the two receptor types with the PDZ scaffold. It was proposed that the regulation of GluN2A-containing NMDAR would involve changes in the pool of receptors within stable nanodomains, while the NMDAR possessing GluN2B subunits are regulated through changes in the nanodomain topography with a stable receptor pool [28, 38]. These findings, together with the evidence showing that GluN2B-MAGUK interactions are only essential for the 1.5 MDa NMDAR supercomplexes [25], suggest that the two pools of receptors belong to distinct protein complexes at hippocampal synapses. Furthermore, since knockout of either PSD95 or PSD93 blocks the assembly of almost all NMDAR-MAGUK supercomplexes of ~ 1.5 MDa, a single MAGUK protein alone does not appear sufficient for the formation of these structures but instead both scaffold proteins are required. In addition, the SH3 domains present in MAGUKs can interact with SH3-binding motifs present in GluN2A and GluN2B [39, 40], but the functional relevance of this interaction remains to be investigated.

The C-terminus of NMDAR subunits contains several sites for post-translational modifications, including phosphorylation, ubiquitination, palmitoylation and acetylation, with impact on the regulation of the receptors. In particular the long C-terminus tail of GluN2 subunits allows multiple mechanisms of regulation of the receptors and triggers a large diversity of intracellular cascades. For example, for the GluN2B subunit that has a particularly long CTD, 11 putative sites for tyrosine phosphorylation were identified, together with 18 sites for serine phosphorylation, and 8 cysteine residues that can undergo palmitoylation [29]. In total, this would comprise a total of 2^37^ possible variants of GluN2B, but the number may be even higher considering the lysine residues that are known to be ubiquitinated but have not been identified yet. The diversity of post-translational modifications (PTM) of NMDAR subunits and the effect on the trafficking and activity of the receptors, as well as on synaptic regulation, will be discussed in the next sections.

## Regulation of NMDA Receptors by Phosphorylation

Phosphorylation of amino acids at the C-terminal tail of NMDAR subunits is the best characterized mechanism of regulation of these receptors. Phosphorylation affects differentially the trafficking and surface distribution of the NMDAR depending on their subunit composition, providing a precise mechanism to control the different receptors depending on the stimuli received by neurons.

**Fig. 1:**
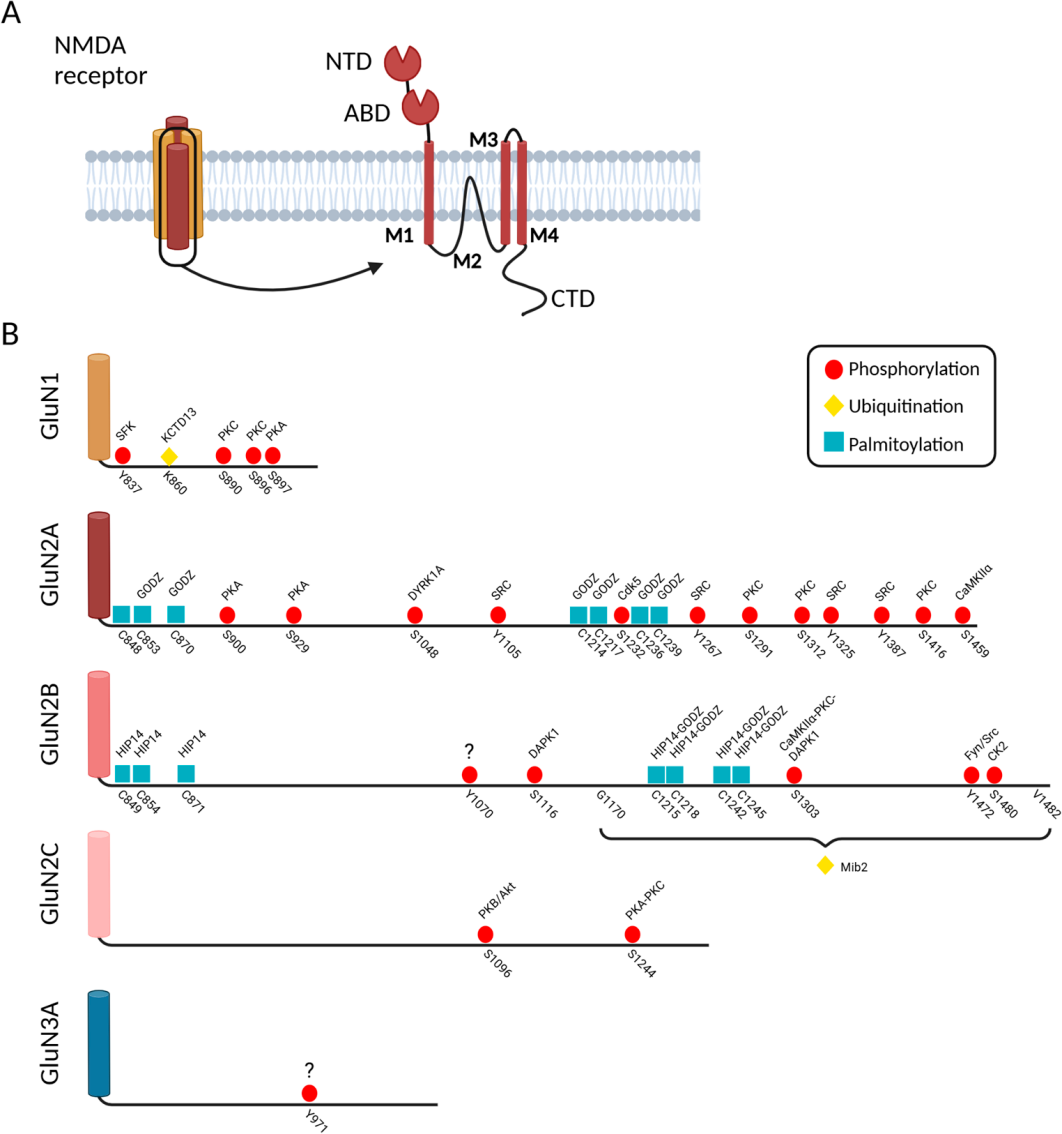
NMDA receptor structure and post-translational modifications (PTMs). (**A**) NMDA receptors (NMDAR) are ligand-gated ion channels composed by four subunits: two obligatory GluN1 subunits and two GluN2 subunits (GluN2A to GluN2D) or two GluN3 subunits (GluN3A, GluN3B). Each subunit is composed by an extracellular region that comprises the N-terminus domain (NTD) and the agonist-binding domain (ABD), by three transmembrane domains (M1, M3 and M4) and a re-entrant loop (M2) that forms the pore of the receptor, and by a intracellular C-terminus domain that shows great diversity among different subunits. GluN1 and GluN3 subunits bind glycine and D-serine, while GliuN2 subunits bind glutamate. (**B**) The C-terminal domain exhibits great difference among different subunits in terms of length and amino acid composition. This domain contains several sites for PTM (mainly phosphorylation, ubiquitination and palmitoylation), here shown together with the enzymes that mediate each PTM. (DHCC3/GODZ and DHCC17/HIP14 have been abbreviated to GODZ and HIP14 respectively for graphical purposes). Created in BioRender. Corti, E. (2025) https://BioRender.com/n56t694

**Fig. 2:**
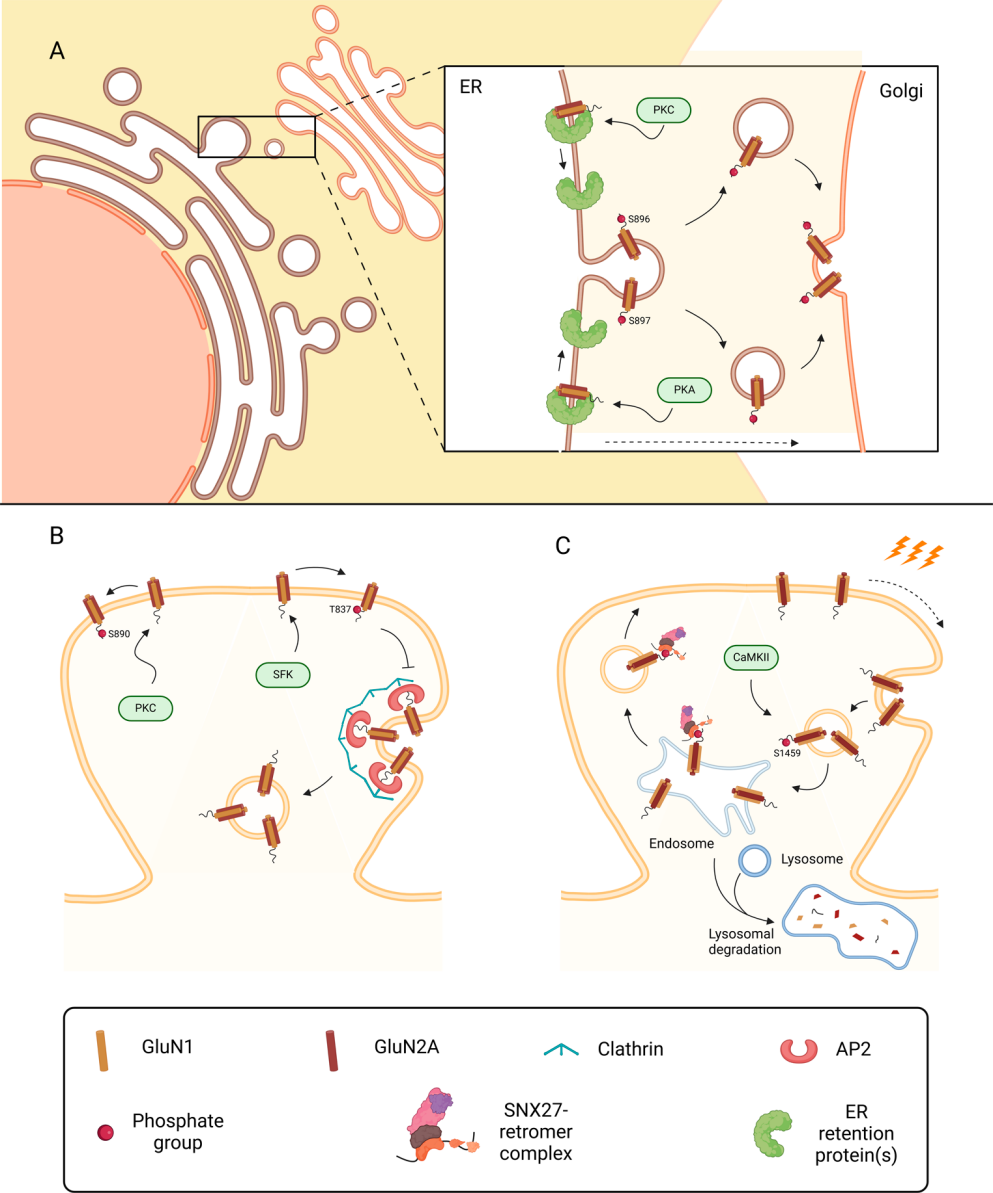
Regulation of GluN1 and GluN2A by phosphorylation. Phosphorylation of amino acids at the C-terminal tail of NMDAR subunits is the best-characterized mechanism of regulation of these receptors, affecting trafficking and surface distribution in a subunit-dependent manner. (**A**) Within the endoplasmic reticulum (ER), phosphorylation of the GluN1 subunit at Ser896 and Ser897 by PKC and PKA, respectively, masks the nearby ER retention motif (Arg-X-Arg), allowing the release of the receptor. Once released, NMDAR traffic through the Golgi apparatus and reach the cell surface. (**B**) At the plasma membrane, PKC-mediated phosphorylation at Ser890 regulates NMDAR surface dynamics. Additionally, Src family kinases (SFK) phosphorylate GluN1 at Tyr837, disrupting its interaction with the AP2 complex, thereby inhibiting clathrin-mediated endocytosis and stabilizing the receptor on the membrane. (**C**) NMDAR internalization and post-endocytic fate depend on their subunit composition. GluN2A-containing receptors are primarily targeted for lysosomal degradation after endocytosis. However, phosphorylation of GluN2A at Ser1459 by CaMKIIα promotes interaction with the SNX27-retromer complex, redirecting the receptor toward recycling instead of degradation. Notably, phosphorylated GluN2A exhibits reduced affinity for PSD-95, suggesting that dephosphorylation may be required before the receptor reintegrates into the synapse. Created in BioRender. Corti, E. (2025) https://BioRender.com/s80x528

The GluN1 obligatory subunit of NMDAR is phosphorylated on Ser896 and Ser897 by protein kinase C (PKC) and protein kinase A (PKA), respectively [41, 42] (Figs. 1B and 2A). Modification of these residues releases the receptors from the endoplasmic reticulum, possibly by masking the neighbour ER retention/retrieval motif (Arg-X-Arg) located in positions 893–895 [43, 44]. After release from the endoplasmic reticulum NMDAR can be found on the cell surface after 2–3 h [43]. PKC also phosphorylates GluN1 on Ser890, but this modification of the receptors appears to play a role in the control of NMDAR dynamics at the cell surface (Fig. 2B) [41, 42]. In addition, phosphorylation of GluN1 CTD on Tyr837 controls the surface expression of NMDAR by regulating the endocytosis of the receptors. Phosphorylation of this residue by Src-family kinases (SFK) disrupts the interaction with the AP2 (clathrin adaptor protein 2) complex, thereby preventing the endocytosis of the receptors [45, 46].

The GluN2A subunit of NMDAR can also be phosphorylated on multiple residues located in the C-terminal tail, providing the opportunity for multiple forms of regulation (Fig. 1B). DYRK1A (dual-specificity tyrosine phosphorylation-regulated kinase 1 A) phosphorylates GluN2A on Ser1048, thereby impairing the internalization of NMDAR and increasing their surface expression [47]. However, the mechanisms whereby GluN2A phosphorylation on Ser1048 affect the internalization of the receptors remain to be investigated. Interestingly, an increased GluN2A expression and prolonged decay of NMDAR-induced calcium transients was observed in cerebellum of TgDyrk1A mice, a mouse model of Down syndrome, suggesting a role for GluN2A phosphorylation in the disease [48].

A major difference between GluN2A- and GluN2B-containing NMDAR is that the former receptors are preferentially targeted for degradation after internalization, whereas the latter pool of receptors are often recycled back to the plasma membrane. The difference arises mainly from the presence of specific CTD motifs close to the TM4 in the GluN1 (Tyr837-Try-Lys) and GluN2A subunits (Tyr842-Try-Lys), which target them for degradation [45, 46]. This mechanism is antagonized by Ca^2+^/calmodulin-dependent kinase IIα (CaMKIIα)-mediated GluN2A phosphorylation on Ser1459 which makes possible the interaction with the sorting nexin 27 (SNX27)-retromer complex, thereby promoting the recycling of the receptors back to the synapse (Fig. 2C) [49]. An increase in GluN2A phosphorylation by CaMKIIα was observed following stimulation of cultured hippocampal neurons with glycine, to model long-term synaptic potentiation (LTP) [49]. Since GluN2A phosphorylated on Ser1459 shows a decreased affinity for PSD-95 [50], the receptor may need to be dephosphorylated before arriving back at the synapse.

In addition to the modulation of receptor trafficking, GluN2A phosphorylation also controls the activity of the receptor. Studies performed in co-cultures of neurons with HEK293 cells transfected with cDNAs encoding human GluN1 and SEP-tagged rat GluN2A subunits, showed that mutations of Ser1459, including the rare S1459G human epilepsy variant, prolong the decay times of NMDAR-mediated currents by increasing the duration of the receptor channel opening [49]. The activity of GluN2A-containing NMDAR can also be controlled by PKA and PKC-mediated phosphorylation. This subunit is phosphorylated by PKA on Ser900 and Ser929, two residues located in the membrane-proximal region of the CTD which is known to control NMDAR desensitization. Accordingly, calcineurin-mediated dephosphorylation or mutation of the two sites enhanced the desensitization and decreased the currents mediated by NMDAR [51–53]. PKC phosphorylates GluN2A in serine residues located closer to the C-terminus (Ser1291 and Ser1312), and this post-translational modification upregulates receptor activity [54]. Furthermore, the PKC-mediated phosphorylation of GluN2A enhances the effects of allosteric modulators of NMDAR, which also bind to extracellular domains, indicating that posttranslational modifications in the C-terminus region of the receptor are coupled to conformational alterations in the extracellular region of the receptor. An additional PKC phosphorylation site on GluN2A, Ser1416, has a local effect to decrease the affinity of this NMDAR subunit for Ca^2+^- and calmodulin-dependent protein kinase II (CaMKII) [55]. Interestingly, CaMKII-mediated phosphorylation of PSD95 on Ser73 results in a decreased interaction of the MAGUK with the GluN2A-CaMKII complex [56].

Phosphorylation of GluN2A by the Src tyrosine kinase (on Tyr1105, Tyr1267 and Tyr1387) impairs the blockade of the receptor channel by Zn^2+^, which is mediated at the NTD of the complex [57]. This observation further suggests that phosphorylation of GluN2A at the CTD induces conformational changes in the receptor with impact in the extracellular domains. The mechanisms by which modification of the GluN2A CTD affects the conformation and function of the extracellular domains remain to be determined. Src-mediated phosphorylation of GluN2A on a different tyrosine residue, located at position 1325, was also proposed to mediate the effects of PAC1 receptors to cause LTP of hippocampal CA1 synapses using conditions of synaptic stimulation that would otherwise produce long-term synaptic depression (LTD) [58]. However, how phosphorylation of this residue affects NMDAR dynamics and/or activity remains to be investigated.

The proline-directed serine/threonine kinase cyclin-dependent kinase-5 (Cdk5) was also shown to phosphorylate the GluN2A C-tail, on Ser1232 [59, 60]. Interestingly, inhibition of the interaction of Cdk5 with GluN2A with a specific peptide prevented the death of CA1 neurons induced by bilaterally occluding common carotid arteries [60]. PSD95 is also a substrate of Cdk5, being phosphorylated in a domain responsible for the interaction with Src [61]. Phosphorylation of PSD95 may regulate the interaction of Src with the NMDAR complex, with impact on the modulation of GluN2A (see above) and Glu2B subunits (see below) by the Src kinase.

**Fig. 3:**
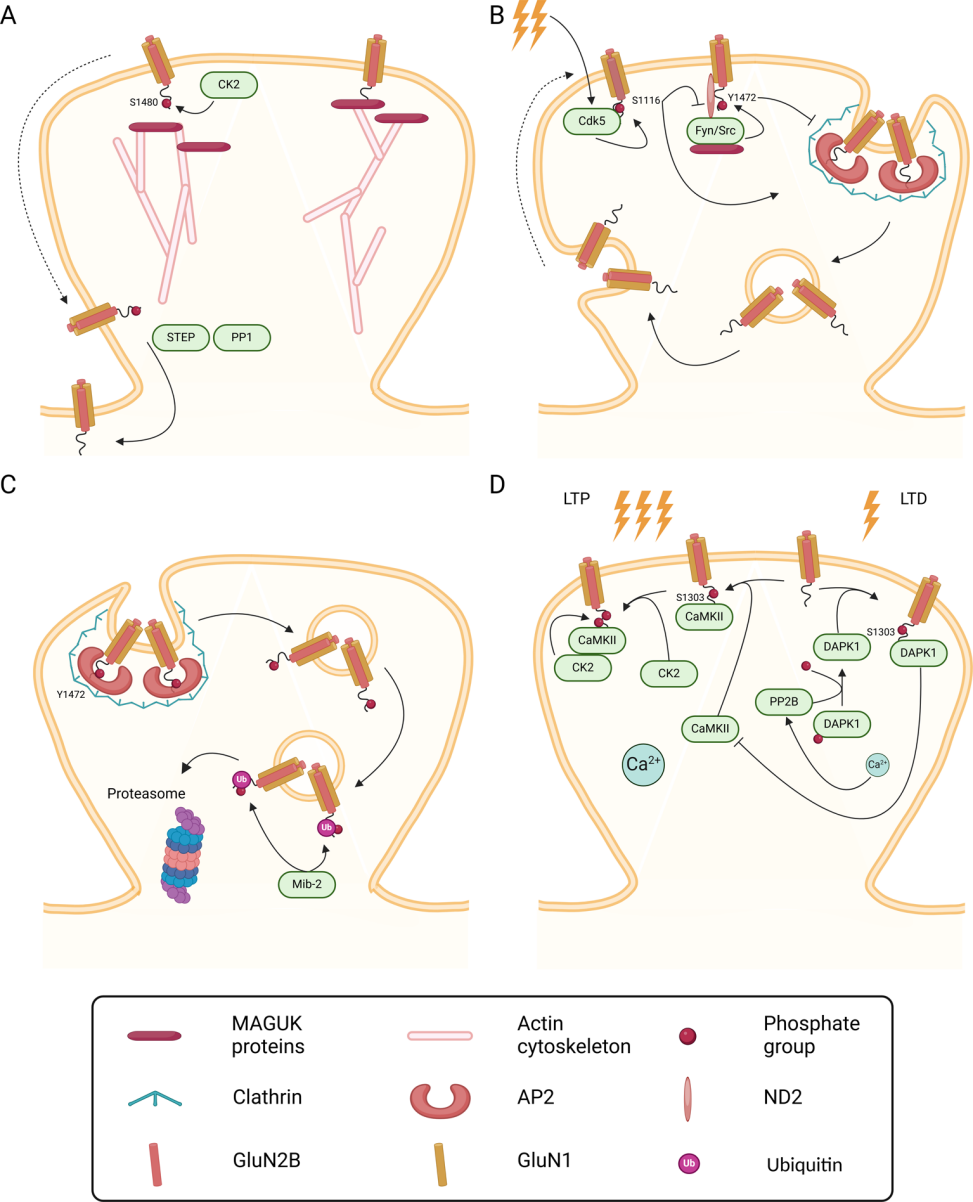
Regulation of GluN2B by phosphorylation. The phosphorylation of GluN2B on different residues by distinct enzymes represents an important strategy to regulate the trafficking and activity of NMDAR. (**A**) GluN2B interacts with MAGUK proteins through its PDZ-binding motif in the C-terminus. This interaction is disrupted upon phosphorylation of Ser1480 by CK2, that decreases the anchoring of the receptor at the synapse. GluN2B-containing NMDAR are then free to diffuse laterally to extrasynaptic sites, where they may be dephosphorylated by STEP or by PP1. (**B**) Upon synaptic activation, GluN2B is phosphorylated by Cdk5 on Ser1116. This promotes the interaction between the consensus tyrosine-based motif (Tyr-Glu-Leu-Lys) and the adaptor complex AP2, followed by clathrin-dependent internalization and recycling to the perisynaptic membrane. At the same time, phosphorylation on Ser1116 by Cdk5 decreases phosphorylation on Tyr1472 by Fyn/Src kinase. Fyn/Src kinase interacts with MAGUK proteins directly and with GluN1 thanks to the mitochondrial-encoded protein ND2. Phosphorylation on Tyr1472 by Fyn/Src kinase, a residue that belongs to the Tyr-Glu-Leu-Lys motif, disrupts the interaction between NMDAR and AP2, thereby decreasing the internalization of the receptor. (**C**) When the interaction between NMDAR and AP2 occurs through a distinct Tyr-Glu-Leu-Lys motif, located closer to the M4 domain in GluN2B, or through GluN1, Tyr1472 can remain phosphorylated. This is a signal for the E3 ligase Mib2 to ubiquitinate GluN2B, targeting the receptor for proteasome-mediated degradation. (**D**) Under conditions of prolonged synaptic activity, such as in LTP, the high Ca^2+^ influx activates CaMKII that phosphorylates GluN2B on Ser1303. Active CaMKII acts as a scaffolding protein for CK2, facilitating the phosphorylation of GluN2B on Ser1480. In conditions of lower Ca^2+^ entry such as LTD, DAPK1 is activated through PP2B-dependent dephosphorylation and phosphorylates GluN2B on Ser1303. This prevents the binding of CaMKII to GluN2B and accumulation within the synapse. Created in BioRender. Corti, E. (2025) https://BioRender.com/d52t992

GluN2B-containing NMDAR can also be regulated by phosphorylation of multiple residues mediated by different protein kinases, which control the trafficking and activity of the receptors (Fig. 1B). Despite the similarity in the amino acid sequence between the C-terminal domain of GluN2A and Glu2B, the mechanisms involved in the regulation of the receptors containing these subunits is quite different. The available evidence indicates that GluN2B phosphorylation is mainly involved in the control of the distribution of NMDAR at the cell surface and their internalization. In particular, phosphorylation on Ser1480 by casein kinase 2 (CK2) occurs within the PDZ ligand domain of GluN2B and therefore decreases the anchoring of the receptors at the synapse by disrupting their interaction with MAGUK proteins (Fig. 3A) [62]. Under these conditions the GluN2B-containing NMDAR are free to diffuse laterally to extrasynaptic sites where they are no longer phosphorylated by protein kinases associated with MAGUKs and may undergo dephosphorylation by striatal-enriched protein tyrosine phosphatase (STEP) [63] or by protein phosphatase 1 (PP1) [64].

Fyn/Src kinases can bind directly to MAGUK proteins, including PSD-95 and SAP102, and are enriched at the PSD [65]; Src also interacts with the GluN1 subunits of NMDAR through the mitochondrial encoded protein ND2 [65]. These tyrosine kinases phosphorylate GluN2B on Tyr1472, a residue that belongs to the consensus tyrosine-based motif (Tyr-Glu-Lys-Leu) recognized by the clathrin adaptor AP2 as part of the mechanism involved in the internalization of the receptors [46, 66–69]. Phosphorylation of GluN2B on Tyr1472 blocks the interaction with the AP2 complex and concomitantly the endocytosis of the receptors (Fig. 3B, C). Therefore, the CK2-mediated phosphorylation of GluN2B on Ser1480 is associated with a decrease in the phosphorylation on Tyr1472 which is followed by internalization of the receptors [70]. Most of these receptors are recycled back to the cell surface after internalization. In contrast, in those receptors that are endocytosed through the action of the first AP2 binding motif present in GluN2B, close to the M4 domain, or by a mechanism mediated by the signal present in GluN1 (at Tyr842), Tyr1472 can remain phosphorylated after internalization and constitute a signal for interaction with the E3 ubiquitin ligase Mind bomb 2 (Mib2) (see below). The resulting ubiquitination of GluN2B CTD tags the receptors for degradation in late endosomes [67, 69].

The network of signalling mechanisms contributing to the regulation of GluN2B-containing NMDAR is influenced by the action of Cdk5, which binds directly to GluN2B and phosphorylates Ser1116 thereby decreasing the surface expression of the receptors in an activity-dependent manner (Fig. 3B) [71]. Phosphorylation of GluN2B on Ser1116 CTD decreases the Src-mediated phosphorylation of the receptor subunit, on Tyr1472, thereby increasing the internalization of the receptors [72]. Importantly, inhibition of the interaction between GluN2B and Cdk5 with a small-interfering peptide facilitated synaptic transmission recorded in cortical pyramidal neurons and improved fear memory [71].

The Fyn/Src-mediated phosphorylation of GluN2B may account for the effects of BDNF to induce the synaptic accumulation of NMDAR in cultured hippocampal neurons [73, 74]. In fact, activation of TrkB (tropomyosin receptor kinase B) receptors for BDNF was shown to activate Fyn with the downstream phosphorylation of GluN2B on Tyr1472 [75]. To what extent these synaptic alterations in GluN2B-containing NMDAR account for the effects of BDNF in LTP of hippocampal synapses [74, 76] has not been investigated. Studies performed with mice with a knock-in mutation of Tyr1472 to a phenylalanine also showed that phosphorylation of this residue is essential for fear learning in experiments using auditory fear conditioning test, and in amygdaloid synaptic plasticity [77]. An attenuation of neuropathic pain was also observed in knock-in mice with a mutation of the Tyr1472 site to phenylalanine [78]. On the other hand, GluN2B phosphorylation on Tyr1070 increases in mice subjected to acute and chronic restraint stress. Experiments with knock-in mice with a mutation on Tyr1070 showed alterations similar to those induced by antidepressants, without affecting cognitive or anxiety related behaviours. Mutation of Tyr1070 also reduced non-synaptic NMDA currents and upregulated the number of glutamatergic synapses in pyramidal neurons of layer 5 of the medial prefrontal cortex [79]. Additional studies are required to elucidate how GluN2B phosphorylation on this site affects the surface distribution of NMDAR.

Another phosphorylation site that is important in the regulation of GluN2B-containing NMDAR is Ser1303, which is a substrate of CaMKIIα and PKC (Fig. 3D) [80]. GluN2B phosphorylation on Ser1303 under conditions of synaptic activity characterized by an increase in the [Ca^2+^]_i_ prevents the interaction with CaMKII which takes places in residues 1290–1310 [81]. Since CK2 binds the active CaMKII [82], the latter kinase can act as a scaffold to facilitate GluN2B phosphorylation on Ser1480 [82] as described above. Therefore, the decreased interaction between GluN2B and CaMKII resulting from the phosphorylation of the NMDAR subunit on Ser1303 has an impact on the phosphorylation of Ser1480 and will ultimately affects phosphorylation of Tyr1472. Together, this indicates that GluN2B phosphorylation of Ser1303, Tyr1472 and Ser1480 is regulated by four different protein kinases which act together in the control of the synaptic expression of NMDAR containing this subunit. This mechanism of GluN2B regulation may contribute to change the GluN2A/GluN2B ratio during sustained synaptic activity, for example during LTP induction [83, 84].

The interaction and regulation of GluN2B-containing NMDAR by death-associated protein kinase 1 (DAPK1) has attracted the interest of the investigators in the field of stroke since a peptide that prevents the interaction of the kinase with GluN2B protected brain damage induced by middle cerebral artery occlusion (MCAO), a model of focal brain ischemia, without affecting the physiological activation of NMDAR [85, 86]. DAPK1 is a Ca^2+^- and calmodulin-dependent serine/threonine protein kinase that interacts with GluN2B in the region between amino acids 1292 and 1304, and phosphorylates the receptor subunit on Ser1303. Interestingly, disruption of the interaction between GluN2B and DAPK1 was also found to have rapid anti-depressant-like effects and reversed chronic unpredictable stress (CUS)-induced alterations in the rat medial prefrontal cortex (mPFC) [87]. Inhibition of DAPK1 also normalized extrasynaptic GluN2B phosphorylation on Ser1303 and surface expression in cortico-striatal neurons, and prevented striatal spine loss in cortico-striatal co-cultures isolated from YAC128 HD mice, a model of Huntington’s disease [88]. Under normal physiological conditions, the regulation of DAPK1-mediated phosphorylation of GluN2B was proposed to play a role in LTD based on the results showing that inhibition of the kinase blocks LTD in CA1 synapses [89]. The low-frequency stimulation required to induce LTD is associated with a low influx of Ca^2+^ and activation of DAPK1 through PP2B-mediated dephosphorylation. Under these conditions, DAPK1 phosphorylates GluN2B on Ser1303, which prevents the binding of CaMKIIα to the receptor subunit and accumulation within the PSD, further enhancing the interaction with DAPK1 [89]. In summary, the Ser1303 phosphorylation site on GluN2B provides the opportunity for integration of multiple signals received by neurons, since it can be regulated by different kinases (PKC, DAPK1 and CaMKII) depending on the physiological context.

GluN2C and GluN2D subunits can also be regulated by phosphorylation, but these mechanisms have been investigated to a lower extent when compared with the modulation of GluN2A and GluN2B-containing NMDAR. These NMDAR subunits possess a shorter C-terminal tail (Fig. 1B) and therefore, it is not surprising that they exhibit a simpler pattern of regulation by protein kinases. PKB/Akt-mediated phosphorylation of GluN2C on Ser1096 upregulates the surface expression of the receptors in cultured cerebellar granule cells following stimulation with IGF-1 or neuronal activation [90]. Phosphorylation of GluN2C on Ser1096 allows the interaction of the receptor with the adaptor protein 14-3-3ε which mediates protein export from the endoplasmic reticulum, followed by an increase in surface expression [90]. In addition, GluN2C can be phosphorylated on Ser1244 by PKA and PKC. Although this phosphorylation site is located adjacent to the consensus PDZ ligand, phosphorylation of GluN2C on Ser1244 does not affect the PDZ interaction and had no impact on the surface distribution of the receptors in an heterologous system. Importantly, NMDAR containing a phosphomimetic mutant of GluN2C exhibited a faster rise and decay of NMDA-evoked currents [91]. However, these observations have not been validated in receptors expressed under more physiological conditions.

In rodents, GluN3A subunits are expressed after birth, with peak levels achieved at the end of the postnatal week. The expression levels of this subunit decrease afterwards, but significant levels of GluN3A are found in the adult brain in basolateral amygdala, ventral hippocampus, paraventricular nucleus, and other ‘high-order’ thalamic nuclei [8, 92–94]. This is distinct from the pattern of expression of GluN3B, which is only found during the postnatal period and in the brain stem and spinal cord [95]. GluN3A- and GluN3B-containing NMDAR differ from the more conventional receptors belonging to this class because they are not activated by glutamate, they display a robust desensitization, very low sensitivity to blockade by Mg^2+^ and reduced Ca^2+^ permeability when compared with the receptors containing GluN1 and GluN2 subunits [8, 92, 93]. Studies performed in mice showed a role for GluN3A-containing NMDAR in the control of internal states, emotional responses and stress-related behaviours [8, 96, 97]. The C-terminus region of GluN3 lacks the PDZ-binding motif present in GluN1 and GluN2 subunits of NMDAR and in fact share few characteristics with these receptor subunits. Therefore, their anchoring to the synapse is not mediated by MAGUKs, but instead require the participation of other scaffold proteins such as plectin [98]. However, the proteins that interact with the GluN3 C-termini largely remain to be investigated [96].

The C-terminal tail of GluN3A contains an endocytic motif (Tyr971-Trp972-Leu973) which binds the AP2 complex for internalization by a clathrin-dependent mechanism. Phosphorylation of the tyrosine residue within this domain (Tyr971 in mice; Tyr980 in humans) leads to internalization of the receptors with a consequent downregulation in their activity [99]. This contrasts with the effect of GluN2B phosphorylation on Tyr1472, which decreases the internalization of NMDAR (see above).

Proteomics studies performed in rat [100, 101] and mice [102] revealed other phosphorylation sites in NMDAR subunits [29], but their role in the regulation of the receptors remains to be investigated.

## Ubiquitination of NMDA Receptors

Ubiquitination of proteins can change their localization, activity and/or stability. Substrate proteins can be modified either by a monoubiquitin, multiple monoubiquitin (multi-ubiquitination) or by polyubiquitin chains (polyubiquitination). In the polyubiquitination of proteins, any of the seven lysine residues (Lys6, Lys11, Lys27, Lys29, Lys33, Lys48, Lys63) of ubiquitin can be linked to the previous one, giving rise to chains with different sizes and configurations [103–105]. The diversity of patterns in protein ubiquitination allows for distinct outcomes in the modified proteins. GluN1, GluN2B and GluN2D subunits of NMDAR were shown to be ubiquitinated, a process that is mediated by E3 ubiquitin-ligase enzymes, following the activity of an ubiquitin-activating enzyme (E1) and an ubiquitin-conjugating enzyme (E2).

**Fig. 4:**
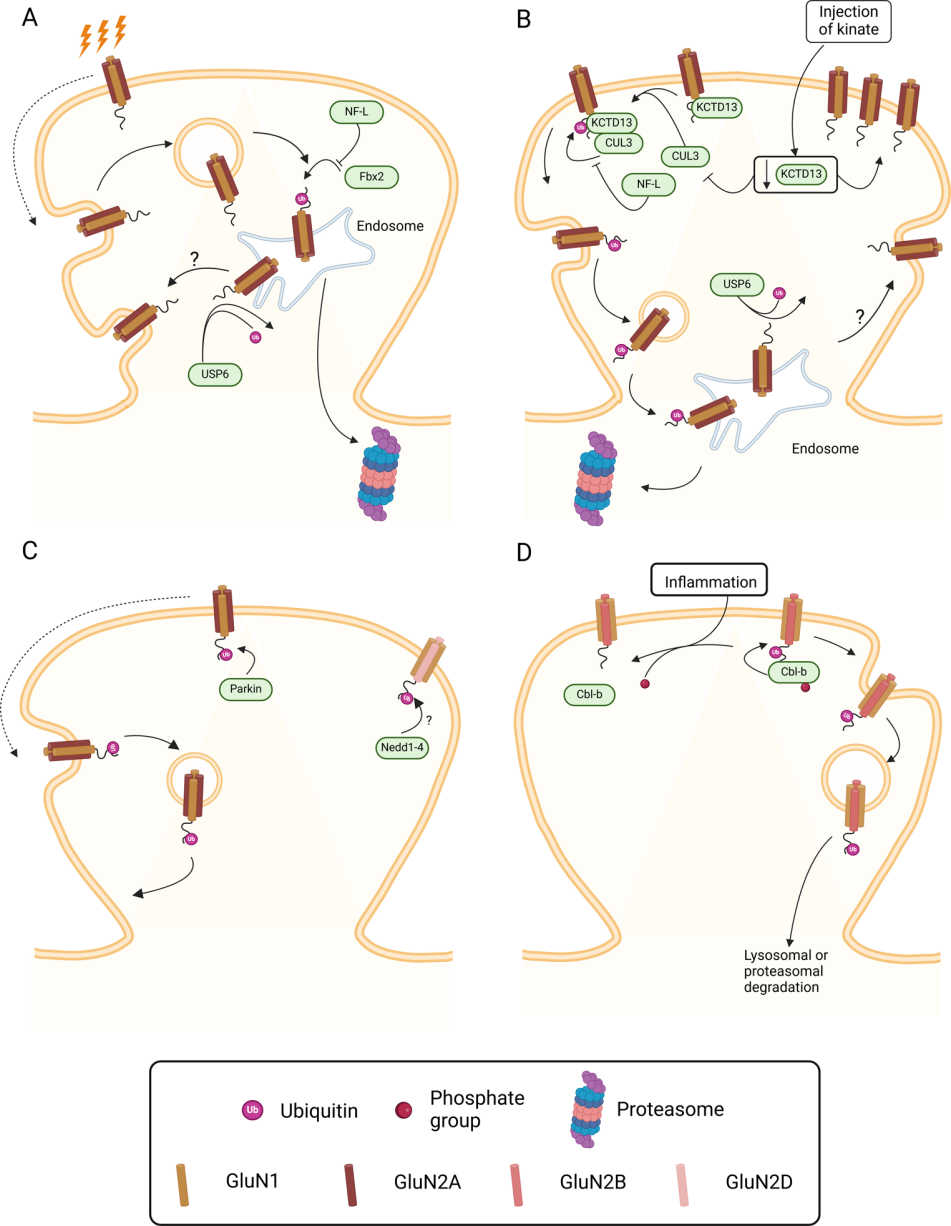
Regulation of NMDAR by ubiquitination. Ubiquitination is a PTM that affects NMDAR location, activity and stability as a consequence of the interaction between the C-terminus tail of NMDAR subunits and different E3 ubiquitin-ligase enzymes. (**A**) For example, following activity-dependent dispersal and internalization of GluN1-GluN2A homodimers, GluN1 is trafficked to the endosome and ubiquitinated by F-box protein 2 (Fbx2). NMDAR are then targeted to proteasome-mediated degradation. (**B**) Another E3 ligase that interacts with GluN1 is KCTD13, that binds to the scaffold protein Cullin 3 (CUL3) and promotes ubiquitination of GluN1 on Lys860 via chains of Lys48-linked ubiquitin. Upon ubiquitination, NMDAR are internalized and trafficked to endosomes, before being targeted for proteasome-mediated degradation. Downregulation of KCTD13 in a model of TLE affects its interaction with CUL3 resulting in a synaptic accumulation of NMDAR. (**A** and **B**) Ubiquitination of NMDAR is also antagonized by neurofilament-L proteins but the molecular mechanism remains unknown. Moreover, GluN1 is a substrate for the deubiquitinating enzyme USP6, that removes ubiquitin chains from target proteins. It was hypothesized that deubiquitinated NMDAR are trafficked back to the surface and re-inserted in the synaptic membrane, but the exact sequence of events is unknown. (**C**) Parkin is an E3 ligase that ubiquitinates GluN1, but this PTM is not associated to degradation of the receptors. Nedd4 is an E3 ligase that ubiquitinates GluN3A but the relevance of this PTM is still unknown. (**D**) In synapses of the dorsal horns in the spinal cord, the E3 ligase Cbl-b interacts with GluN2B and ubiquitinates this subunit by adding Lys63-linked ubiquitin chains. This promotes internalisation and proteasome- or lysosome-mediated degradation of NMDAR. Injection of complete Freund’s adjuvant triggers peripheral inflammation that leads to a dephosphorylation of Cbl-b and a decrease in the interaction between Cbl-b and the C-terminus of GluN2B. Created in BioRender. Corti, E. (2025) https://BioRender.com/r30q768

The F-box protein Fbx2 (F-box only protein 2) belongs to the SCF (Skp1-Cullin-F-box) complex and binds to high-mannose glycans present in the extracellular domain of GluN1. It was proposed that GluN1 ubiquitination through this pathway plays a role in the degradation of NMDAR that are internalized following activity-dependent dispersal of the receptors away from the synapse (Fig. 4A). This mechanism of regulation of NMDAR is limited to the population of receptors containing GluN2A subunits since studies performed in *Fbxo2* KO mice showed no alterations in GluN2B protein levels in the hippocampus [106]. Interestingly, Fbx2 is associated with endoplasmic reticulum associated degradation (ERAD) of proteins [107], a pathway involved in the removal of misfolded proteins and orphan subunits that exit the endoplasmic reticulum. This suggests that ERAD may also play a role in the regulation of NMDAR after the activity-dependent internalization from the neuronal surface. *Fbxo2* KO mice also exhibited an upregulation in the number of axo-dendritic shaft synapses, without affecting dendritic spine density and hippocampal synaptic transmission and plasticity, suggesting a role for the SCF complex in the control of synapse formation [106]. The regulation of GluN1 by ubiquitination may be subjected to modulation by interaction with neurofilament-L (NF-L) proteins (Fig. 4A, B). Indeed, studies performed in *NFL* KO mice showed an increase in K48-linked GluN1 ubiquitination in hippocampal synaptic fractions, and a decrease in total GluN1 protein levels [108]. The Lys48 polyubiquitin chain is the main form of ubiquitin modification involved in protein degradation by the ubiquitin-proteasome system [105]. The regulation of GluN1 by Fbx2 may also be relevant in the response to stress since an upregulation of this NMDAR subunit was observed in the prefrontal cortex of mice subjected to repeated stress, and the effect was blocked using an RNAi against Fbxo2 as well as by administration of glucocorticoid receptor antagonists [109].

A recent study identified KCTD13 (potassium channel tetramerization domain 13) as a novel E3 ligase substrate adaptor important in the regulation of the abundance of GluN1. KCTD3 binds to the scaffold protein CULLIN3 (CUL3), a RING domain protein, to ubiquitinate GluN1 thereby promoting the degradation of the NMDAR at the proteasome (Fig. 4B). Lys860 was identified as the main site for GluN1 ubiquitination mediated by KCTD13, and the ubiquitin chains were Lys48-linked. As expected from these findings, downregulation of KCTD13 increased the amplitude of NMDAR-mediated currents in hippocampal synapses [110]. A decrease in the KCTD13 protein levels was also observed in the brain tissue from patients with temporal lobe epilepsy (TLE). The KCTD13 protein levels in the hippocampus were dynamically changed in the mice subjected to hippocampal injection of kainate, used as a model of TLE, but were significantly decreased in the chronic phase characterized by an hyperexcitability of the abnormal neuronal networks [110]. Additional studies are required to elucidate how KCTD13 and Fbx2 cooperate to control the degradation of NMDAR by regulation of GluN1 subunits.

The E3 ligase Parkin, which is associated with Parkinson’s disease (PD), may also play a role in the regulation of GluN1 by ubiquitination (Fig. 4C). Indeed, overexpression of Parkin in non-neuronal cells increased GluN1 ubiquitination, an effect that was impaired in PD-associated mutant forms of the protein. Studies performed in cultured hippocampal neurons showed that Parkin-mediated GluN1 ubiquitination is not coupled to the degradation of NMDAR but instead are important in the internalization and recycling of the receptors [111]. However, the downregulation of surface GluN1 observed in studies with transfected cultured neurons, which showed a role for Parkin in the maintenance of surface GluN1, contrast with the results obtained in experiments with Parkin KO mice, which showed the opposite effects [112]. These differences may be due to the use of different models, but additional studies are required to elucidate the regulation of GluN1 by Parkin.

Deubiquitinating enzymes counter the signal induced by E3-ligases by removing ubiquitin from these substrates, thereby decreasing their degradation in the proteasome. GluN1 is a substrate of deubiquitinate USP6 (ubiquitin-specific protease 6) (Fig. 4B) and studies performed in transgenic mice overexpressing the human enzyme in the cerebral cortex and hippocampus showed a downregulation in NMDAR ubiquitination, associated with an increase in the abundance of NMDAR subunits in the cerebral cortex (GluN1, GluN2A and GluN2B) and hippocampus (GluN1 and GluN2B), and in the surface expression of GluN1 in cultured neurons isolated from the same animals. This upregulation in the surface expression of NMDAR was correlated with an increase in LTP and a decrease in LTD in hippocampal CA1 synapses. In addition, downregulation of USP6 in cultured neurons derived from human embryonic stem cells impaired the clustering of NMDAR at the postsynaptic densities and decreased synaptic function [113].

The GluN2B subunit of NMDAR can also be regulated by ubiquitination, mediated in this case by the E3 ligase Mind bomb-2 (Mib2) (Fig. 3C). Interaction of this enzyme with the GluN2B cytoplasmic tail (between amino acids 1170 and 1482), and the ubiquitination of the NMDAR subunit, depend on the phosphosphorylation of GluN2B by the tyrosine kinase Fyn. GluN2B ubiquitination is followed by degradation of the NMDAR by the proteasome, with a consequent reduction in the receptor activity [69]. These results correlate with the observed impairment in the early- and late-phases of hippocampal LTP in Mib2 knock-out mice [114]. Surprisingly, no alterations were observed in the basal synaptic transmission and in LTD in hippocampal CA1 synapses in Mib2 knock-out mice, as well as in the total levels of GluN2B. However, whether there were alterations in GluN2B ubiquitination in the hippocampus of Mib2 knock-out mice was not investigated. The observed upregulation in GluN2B protein levels in the cerebral cortex and hippocampus of transgenic mice overexpressing the human USP6 suggests that this enzyme plays an important role in the control of the rate of GluN2B-containing NMDAR degradation by regulating the growth of polyubiquitin chains [113].

Neurons of the spinal cord dorsal horn are enriched in the E3 ubiquitin ligase casitas B-lineage lymphoma b (Cbl-b), which also regulates GluN2B-containing NMDAR by ubiquitination. It was proposed that Cbl-b interacts with GluN2B through its N-terminal tyrosine kinase binding domain to limit the synaptic abundance of NMDAR containing this subunit in adult mice (Fig. 4D). Induction of peripheral inflammation in mice through intraplantar injection of complete Freund’s adjuvant led to a dephosphorylation of Cbl-b with a consequent decrease in the interaction of the ubiquitin ligase with the GluN2B C-terminal tail, and reduction in ubiquitination of the target protein. Together, this work showed that the composition of NMDAR in dorsal horn synapses is controlled by ubiquitination, and peripheral inflammation impairs these mechanisms, thereby enhancing nociception [115]. Cbl-b is also concentrated in synaptic regions at the CA1, CA3 and dentate gyrus in the hippocampus and *cbl-b* null mice exhibit an enhancement in short-term plasticity and increased long-term memory [116]. However, to what extent Cbl-b also controls the dynamics of GluN2B-containing NMDAR in the hippocampus has not been investigated.

A biochemical study also identified an interaction between the E3 ligase Nedd4 (neural precursor cell-expressed developmentally downregulated) and the C-terminal region of GluN2D [117] (Fig. 4C), but the role ubiquitination in the regulation of NMDAR containing this subunit under physiological conditions remains to be investigated.

Studies performed in cultured hippocampal neurons showed that neuronal activity is coupled to the activation of the proteasome and induce a redistribution of the proteasomes from the dendritic shaft to dendritic spines. This redistribution of the proteasomes is mediated by CaMKII$\:\alpha\:$-mediated phosphorylation of the Rpt6 proteasome subunit [103, 118]. This redistribution may facilitate the degradation of NMDAR by the proteasome following internalization.

## Regulation of NMDA Receptor Function by Palmitoylation

S-palmitoylation is a posttranslational modification of proteins consisting in the covalent attachment of the lipid palmitate, a 16-carbon saturated fatty acid, to the SH group of target proteins through thioester bonds. This is a reversible reaction catalysed by palmitoyl acyl transferases (PAT) [119], being depalmitoylation mediated by palmitoyl-protein thioesterases (PPT) [120].

**Fig. 5:**
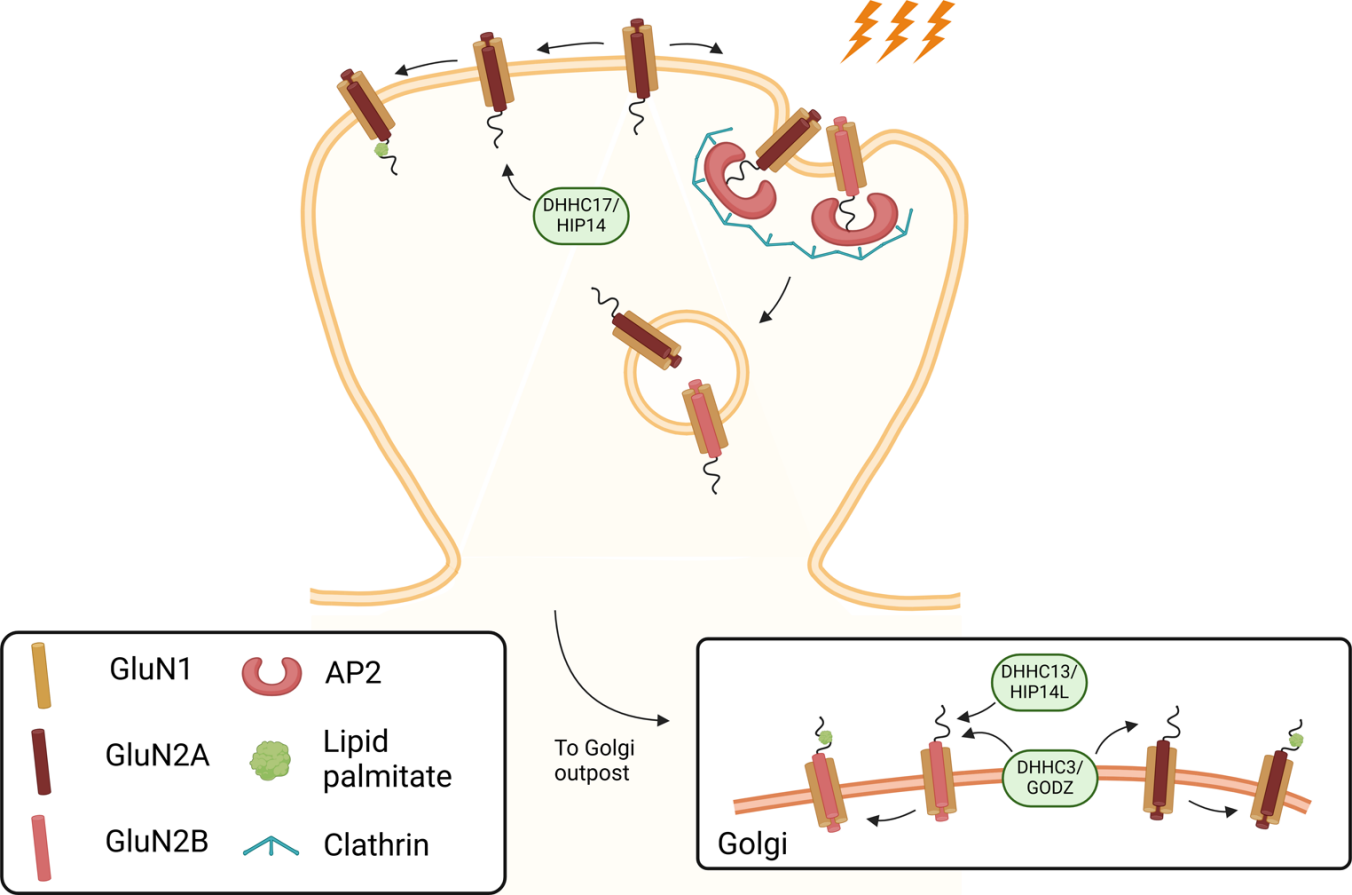
Regulation of NMDA Receptor Function by Palmitoylation. Palmitoylation is a post-translational modification that involves the attachment of palmitate to cysteine residues, influencing protein trafficking and surface expression. In GluN2A and GluN2B NMDAR subunits, palmitoylation occurs at two cysteine clusters within their C-terminal tails. Cysteine cluster I, located near the plasma membrane, regulates receptor internalization, while cysteine cluster II, positioned in the middle of the C-terminal tail, is associated with palmitoylation in the Golgi apparatus and affects receptor trafficking. DHHC17/HIP14 mediates palmitoylation at cluster I in GluN2B, impacting receptor retention, while DHHC3/GODZ targets cluster II in both subunits, influencing receptor trafficking. DHHC14/HIP14L also contributes to GluN2B palmitoylation. Created in BioRender. Corti, E. (2025) https://BioRender.com/g25p911

The GluN2A and GluN2B NMDAR subunits (but not GluN1 subunits) are both palmitoylated in one or more cysteine residues belonging to two cysteine clusters located in their C-terminal tail. The Cysteine cluster I is located next to TM4 of both subunits, and includes Cys848, Cys853 and Cys870 in the case of GluN2A, and Cys849, Cys854 and Cys871 in GluN2B [121]. DHHC17/huntigtin-associated protein 14 (HIP14) is the only PAT identified so far as mediator of palmitoylation of GluN2B cysteine cluster I (Fig. 5). DHHC3/GODZ, a Golgi apparatus-specific protein, mediate the palmitoylation of Cysteine cluster II located in the middle of the C-terminal tail of GluN2A and GluN2B, and HIP14L also targets the same cysteine residues in GluN2B [122, 123].

The palmitoylation state of the GluN2A and GluN2B subunits in the Cysteine cluster I located next to the plasma membrane controls the surface expression and internalization of both types of receptors (Fig. 5). In particular, it regulates the constitutive internalization of NMDAR during development, possibly by affecting the Fyn-mediated phosphorylation of tyrosine-based internalization motifs present in the C-terminus tails of GluN2 subunits [46]. These motifs are adjacent to Cysteine cluster I and are necessary and sufficient for internalization of NMDAR by allowing the interaction with the µ2 subunit of the AP2 complex (see above). Therefore, GluN2 palmitoylation on the Cysteine cluster I decreases the clathrin-mediated internalization of the receptors, thereby enhancing their surface expression [122]. In particular, the GluN2B subunit C-terminus tail contains the motif Tyr1472-Glu-Lys-Leu, which is the major target of Src kinases. Activity-dependent phosphorylation of GluN2B on Tyr1472 by Fyn prevents clathrin-mediated internalization of these receptors [67, 68], and controls the synaptic distribution of GluN2B-containing NMDAR in hippocampal neurons [67] and in the amygdala [77]. Mice with mutations in the GluN2B Cysteine cluster I showed a reduction in GluN2B phosphorylation in Tyr1472 in the hippocampus and cerebral cortex [124], which is coupled to receptor internalization [68]. Interestingly, these mice were characterized by a limb-clasping response showing a role for palmitoylation of NMDAR in complex higher brain functions [124].

The Cysteine cluster I present in GluN2B also plays an important role in the Ca^2+^-induced inhibition of NMDAR by endogenous neurosteroids. Studies performed in cultured hippocampal neurons and in an heterologous system showed that an increase in [Ca^2+^]_i_ leads to the depalmitoylation of GluN2B C-terminal tail at the Cysteine cluster I thereby enhancing the sensitivity to inhibition by neurosteroids, which reduce the probability of channel opening [125].

The depalmitoylating enzyme palmitoyl-protein thioesterase 1 (PPT1) was shown to play an important role in the surface retention of GluN2B-containing NMDAR and in development in the mouse visual cortex [126]. These observations point to an important role for this enzyme in the control of GluN2B palmitoylation levels. The GluN2C and GluN2D C-terminal tails are shorter, when compared with GluN2A and GluN2B, but share homology with one and two palmitoylated cysteines, respectively [122]. However, it remains to be determined whether NMDAR containing these subunits are also regulated by this mechanism.

Interestingly, alterations in the regulation of NMDAR by palmitoylation have been shown to play a role in diseases of the nervous system. Studies performed in neuronal cultures prepared from YAC128 mice, a model to study Huntington’s disease, showed a decrease in the HIP14L-mediated palmitoylation of GluN2B, specifically on Cysteine cluster II, which was associated with an upregulation in extrasynaptic GluN2B-containing NMDAR in striatal neurons. It was proposed that alterations in GluN2B palmitoylation levels by HIP14L may contribute to activate cell death-signaling pathways in Huntington’s disease, leading to striatal atrophy and motor impairment [123]. Mutations in the depalmitoylating enzyme PPT1 are associated with infantile ceroid lipofuscinosis (CLN1), a paediatric neurodegenerative disorder. As expected, studies performed with *Ppt1*^*−/−*^ mice showed an upregulation in GluN2B palmitoylation associated with alterations in dendritic spine morphology, which may underlie CLN1 [126]. Whether changes in NMDAR palmitoylation contribute to neuropsychiatric diseases remains to be investigated.

## Concluding Remarks

NMDAR are very dynamic and their distribution at the synapse and in extrasynaptic locations is determined by interaction with multiple binding partners and regulated post-translational modifications. These multiple levels of regulation play a key role in the control of the numerous functions played by NMDAR, under normal physiological conditions and in diseases of the nervous system. The available evidence shows a differential nanodomain distribution of GluN2A- and GluN2B-containing NMDAR at the synapse [27, 28], and in nanocolumns adjacent to pre-synaptic glutamate release sites [24]. Additional superresolution imaging studies [38] are required to determine how this differential distribution of NMDAR receptors is regulated by post-translational modifications. The diversity of synaptic responses mediated by NMDAR discussed in this article is further increased considering that their activity depends on the depolarization of the membrane resulting from the activation of AMPA receptors, which are also regulated by post-translational modifications [127]. In addition, differences may be expected between distinct brain regions depending on the local composition of the synaptic proteome.

The study of the regulation and function of NMDAR has been largely focused on GluN2A and GluN2B-containing NMDAR, which are highly expressed in the cerebral cortex and in the hippocampus, and have been implicated in neurological disorders. Future research should also improve the knowledge in the specific mechanisms controlling the distribution and function of NMDAR with other subunit compositions. Differences in their C-terminal tail suggest a differential interaction with other binding partners important in the control of NMDAR endosomal trafficking, supercomplex formation and synaptic plasticity. Proteomics studies have been instrumental along the years in the identification of glutamate receptor binding partners that control their intracellular trafficking and surface distribution (e.g [25, 26]), and may also contribute to the identification of novel posttranslational modifications of NMDAR as well as new interactors. Fluorescence imaging studies together with electrophysiology experiments are widely used tools to investigate the functional implications of the interaction of NMDAR with specific proteins, and the influence of posttranslational modifications (see above for references).

The analysis of the functional role of specific post-translational modifications in NMDAR subunits is typically performed in mutant mice, in which specific amino acids are mutated to impair the mechanism under investigation and analyze the impact on plasticity and cognition. However, given the diversity of post-translational modifications involved in the regulation of NMDAR, and the possibility of combining several modifications in a single receptor subunit, additional studies using novel mutant mice are required to better understand how post-translational modifications can affect synaptic function, plasticity mechanisms and cognition. In vivo studies should also contribute to understand how post-translational modifications of NMDAR are coupled to the multiple forms of plasticity and are coordinated in cognitive responses. Additional studies are also required to better understand how the impairment of NMDAR function is coupled to neuropsychiatric and neurodegenerative disorders.

## Data Availability

No datasets were generated or analysed during the current study.
